# Severe hypothermia in transported pullets: case study of its occurrence, diagnosis and treatment using active external rewarming technique

**DOI:** 10.1002/vms3.62

**Published:** 2017-03-10

**Authors:** Ndazo S. Minka, Joseph O. Ayo

**Affiliations:** ^1^ College of Agriculture and Animal Science Division of Agricultural Colleges Ahmadu Bello University Mando‐Kaduna Nigeria; ^2^ Department of Physiology Faculty of Veterinary Medicine Ahmadu Bello University Zaria Nigeria

**Keywords:** behaviour, blood biochemistry, colonic temperature, pullets, rewarming, severe hypothermia

## Abstract

Sixty pullets, aged 17 weeks, were presented when presumed to be ‘dead’ after being exposed to wet‐cold weather transportation. The birds appeared unconscious and their feathers were soaking wet, and with a body rigid and cold to touch. The aim of the study was to resuscitate the hypothermic pullets. Blood samples were obtained and core body temperature recorded before and after rewarming. The birds were resuscitated using active external rewarming technique. Blood samples revealed significant (*P *<* *0.05) decreases in the concentrations of serum electrolytes of Na, Cl, K, Ca and P; and renal function and activities of the serum enzymes of AST, ALT, ALP and CRT decreased compared to baseline reference normal values. Colonic temperature, recorded through the cloacae, revealed a temperature of 29 ± 0.4°C. The presumptive diagnosis was severe hypothermia. Treatment of the pullets using active external rewarming technique for 7 to 10 h resulted in successful restoration of all the behavioural, biochemical and colonic temperature responses to normal values. The treatment resulted in a complete recovery of all the birds with no signs of illness at 4‐week follow‐up. To the best of our knowledge, this study is one of the first reports to evaluate the behavioural and biochemical responses of pullets accidentally exposed to severe hypothermia, and successful treatment of the birds using active external rewarming technique.

## Introduction

Accidental hypothermia is defined as an unintentional fall in core temperature to <35°C (Mallet 2002; Brown *et al*. 2012). Livestock are often exposed to accidental hypothermia during natural disasters, like flooding (Johnson and Muller 2002) and during road transportation under chill wind (Hunter *et al*. 1999). Over 2 000 000 chickens were reported to have died as a result of flooding during Hurricane Floyd in North Carolina in 1999, and hypothermia is the most frequently reported illness of all animals that are rescued from flood waters (Johnson *et al*. 2002).

Transportation of matured live birds by road is an inevitable component of the poultry industry. Unfortunately, transportation induces severe stress in birds; which may cause their suffering or death, especially when it is done under extreme and/or unethical conditions (Minka & Ayo 2008, 2010; Burlinguette *et al*. 2011). Heat and cold are two major stress factors contributing to both death and overall adverse effects of road transportation stress in poultry (Minka & Ayo 2008; Knezacek *et al*. 2010; Burlinguette *et al*. 2011).

Information on hypothermia in poultry is grossly inadequate in the available literature and very little is known about the impact of cold exposure on poultry welfare during transport. Besides, the mechanisms underlying the pathophysiological responses of birds to rewarming following accidental transportation hypothermia have not been elucidated. This is not surprising because overwhelming majority of birds transported worldwide are broilers, and are only destined for slaughter on arrival. In addition, most veterinary clinics and emergency disaster shelters for animals lack units and equipment that handle severe hypothermic cases in poultry.

This study demonstrated biochemical and behavioural changes, occurring in severely hypothermic pullets, and the effectiveness of active external rewarming technique in resuscitating the pullets to life.

## Materials and methods

### Case report

Sixty 17‐week‐old ‘presumed dead’ pullets were presented to a Farm Veterinary Clinic at 01:00 am. The pullets were unconscious and lifeless, with soaking wet feathers. On admission to the clinic, the birds were kept in a well‐illuminated, clean, dry and warm compartment, under 30°C.

These 60 birds were among the 920 apparently healthy Shika Brown pullets that were undergoing normal commercial transportation as part of an experimental setup**.** In the planned experimental setup, the physiological variables of the birds were to be assessed before and after transportation periods. Consequently, the baseline physiological values obtained in the experimental setup were used as part of the history of the case of this study. During the transportation, heavy rainfall was encountered unexpectedly and resulted in the soaking of 60 pullets that were located close to air inlets of the vehicle. The wetting of the 60 birds by the rain was accidental, and not part of an experiment**.** The 60 birds were collected after offloading from drawers that were directly affected by the air inlets at the sides and rear planes of the vehicle.

### Behaviour and core body temperature measurements

On admission to the clinic, the birds’ physical appearance and behaviour were visually analysed. The colonic temperatures of the birds were recorded by inserting a thermistor probe (Yellow Spring Instruments, Model 46 TUC, Yellow Spring, OH) into the cloaca of each pullet to a depth of 4–5 cm.

### Blood sampling and analysis

Blood samples for the analysis of serum electrolytes and enzymes were obtained from 30 birds selected at random. Exactly 3 mL of blood was collected from the wing vein of the birds into non‐heparinised sterile tubes and centrifuged at 9.500 g for 15 min to obtain serum. The serum obtained was used for the determination of concentrations of electrolytes (sodium, potassium, chloride, calcium, phosphorus and bicarbonate), metabolites (creatinine and urea), and activities of enzymes [aspartate aminotransferase (AST), alanine aminotransferase (ALT) and alkaline phosphatase (ALP)]. Serum concentrations of sodium, calcium, phosphorus and potassium were determined using plasma atomic emission spectrophotometric kits (Glaxo and Wipro, India) and chloride by means of chloride meter (Jenway PCLM3, Essex, UK). Standard commercial kits (Glaxo and Wipro, India) were used to determine AST, ALT and ALP activities, and urea and creatinine (CRT) concentrations as per manufacturer's instructions.

### Rewarming of the pullets

We hypothesized that rewarming the pullets as soon as possible would be beneficial in restoring their physical appearance, behaviour, biochemical values and body temperature to normal. This study employed active external rewarming for the treatment of severely hypothermic pullets as described by Minka & Ayo (2016). Briefly, the active external rewarming was performed using a calibrated electrical brooder heater plate, heated to a temperature of about 45–48°C. The rewarming temperature was extrapolated from human medicine (Connolly & Worthley 2000), taking into consideration the higher normal CBT of chicken, which ranges from 40 to 42°C. Before rewarming, all visible water was removed from the feathers and body of each bird by gentle patting with a warm hand towel; thereafter, the pullets were exposed to the hot plate. Firstly, the rewarming of each pullet was done by exposing the chest and under the wing areas (areas with major blood flow) to the hot plate for a period of 1–2 min, followed by the sides and later the extremities for the same period, then a break of 2 min. The technique was repeated several times until the CBT of the pullets attained the normal minimum value of 40°C. The behaviour and CBT of the pullets were recorded during each hour of the rewarming period, while blood sampling was repeated after their CBT attained the normal minimum value of 40°C. During the clinical period, the pullets were gently and humanely handled, and the gradual pattern of rewarming used in this study followed the recommended technique of rewarming hypothermic humans (European Resuscitation Council 2010).

A day (24 h) after the treatment, the pullets were colour‐marked for easy identification and raised on deep litter in a general pen. Thereafter, the behaviour, CBT and biochemical values of the pullets were evaluated bi‐weekly for 4 weeks.

### Statistical analysis

Data were analysed using the SAS system (SAS Institute Inc., Cary, NC). All data are expressed as mean ± SEM. Data were normally distributed (*P *<* *0.05, Kolmogorov–Smirnov test). One‐way repeated‐measures ANOVA was used to determine the statistically significant effects of study conditions (baseline, hypothermic and during rewarming period). Values of *P *<* *0.05 were considered significant. Bonferroni's multiple comparison test was applied for post hoc comparison.

## Results

### Behaviour and core body temperature

Table 1 shows the effects of severe hypothermia and 7 h of gradual active rewarming on the physical appearance, behaviour and CBT of the hypothermic pullets. The hypothermic birds appeared lifeless, unconscious with eyes closed, cold and rigid body. Their feathers were heavily soaked with cold rain water. The legs and neck were distended and inflexible.

**Table 1:** Core body temperature and behavioural responses of severely hypothermic pullets to 7 h of active external rewarming (*n* = 30)

Rewarming hour (h)	Core body temperature (^°^C)	Appearance/Behaviour
0	28.0 ± 0.2	Lifeless, unconscious, wet and soaked feather, rigid and cold body to touch
1	29.0 ± 0.5	Stretching of legs and wings
2	36.0 ± 0.2	Blinking of eye, beak movement, recumbency
3	36.5 ± 0.1	Recumbency, eye open, beak opening
4	37.0 ± 0.3	Recumbency, conscious, response to stimuli
5	37.2 ± 0.5	Recumbency, neck and head erect. Standing and staggered, hanged feathers
6	38.9 ± 0.2	Standing and walking reluctantly, huddling in small groups
7	40.1 ± 0.1 Stoppage of rewarming	Commencement of feeding
8	40.2 ± 0.4	Active movement and feeding
9	40.5 ± 0.2	Normal
10	41.5 ± 0.2	Normal

Normal = lively, active movement, feeding/foraging or preening and dust bathing.

The CBT recorded from the hypothermic pullets admitted to the clinic before rewarming had minimum, maximum and mean values of 28, 30 and 29 ± 0.4°C, respectively, which were significantly (*P *<* *0.001) lower than the baseline values of 40.2–41.8°C. Based on the physical appearance, behaviour, CBT and biochemical evaluations, our main diagnosis was severe hypothermia.

During rewarming of the pullets, the CBT values increased sharply and progressively, with no sign of ‘afterdrop’, a continued decline of CBT. Normothermia was achieved after 7 h of gradual rewarming. Severely hypothermic pullets were able to stand upright on their legs and walked slowly after 6 h of active external rewarming. Feeding and foraging behaviours were returned to normal from the 7th hour of gradual rewarming. The pullets returned to normal behaviours, which constituted fast movement, preening and dust bathing, from the eighth to the 10th hour after admission to the clinic (Table 1).

### Blood analysis

**Figure 1:**
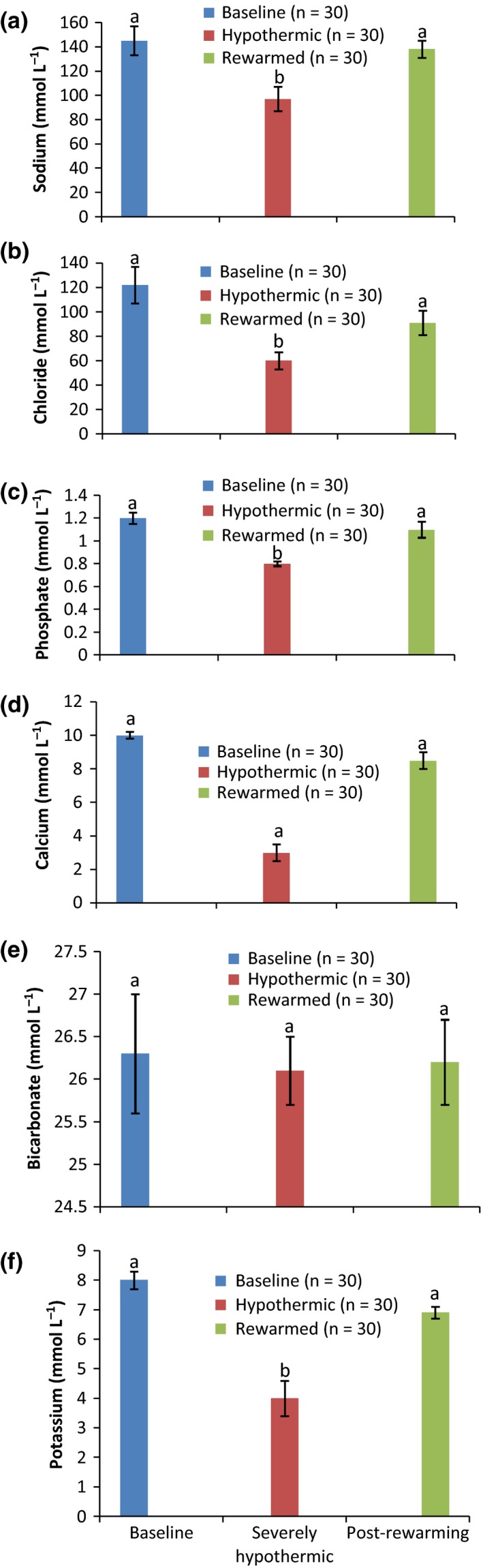
Concentrations of serum electrolytes in normal, severely hypothermic and rewarmed pullets.

**Figure 2:**
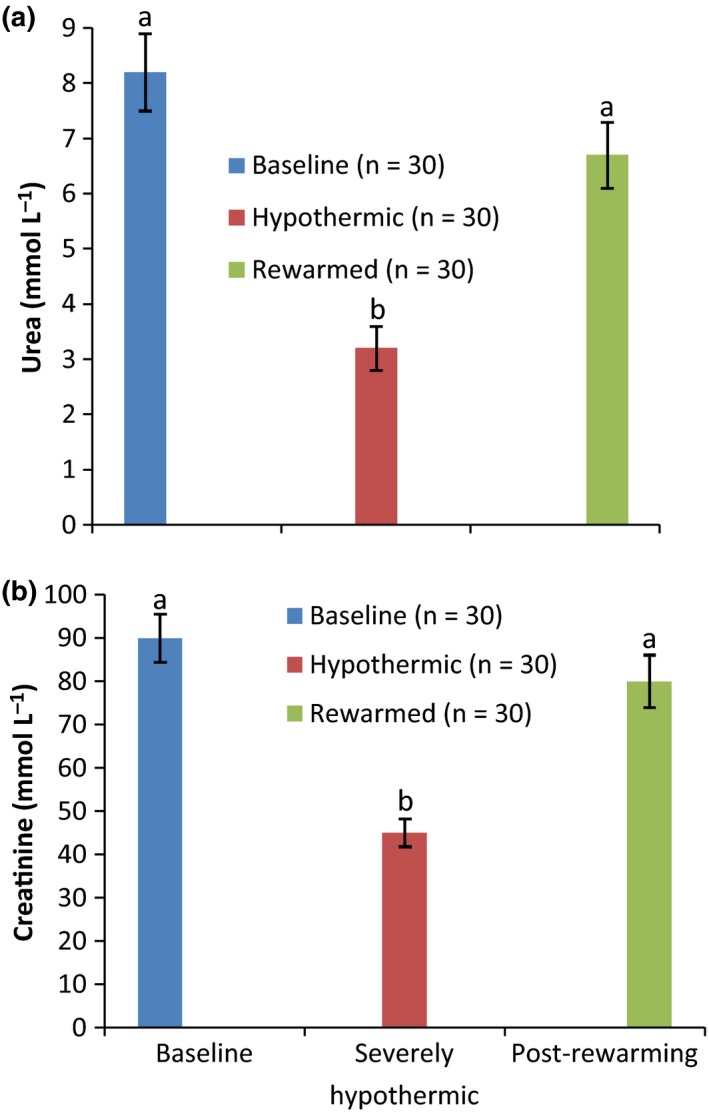
Metabolites of normal, severely hypothermic and rewarmed pullets.

**Figure 3:**
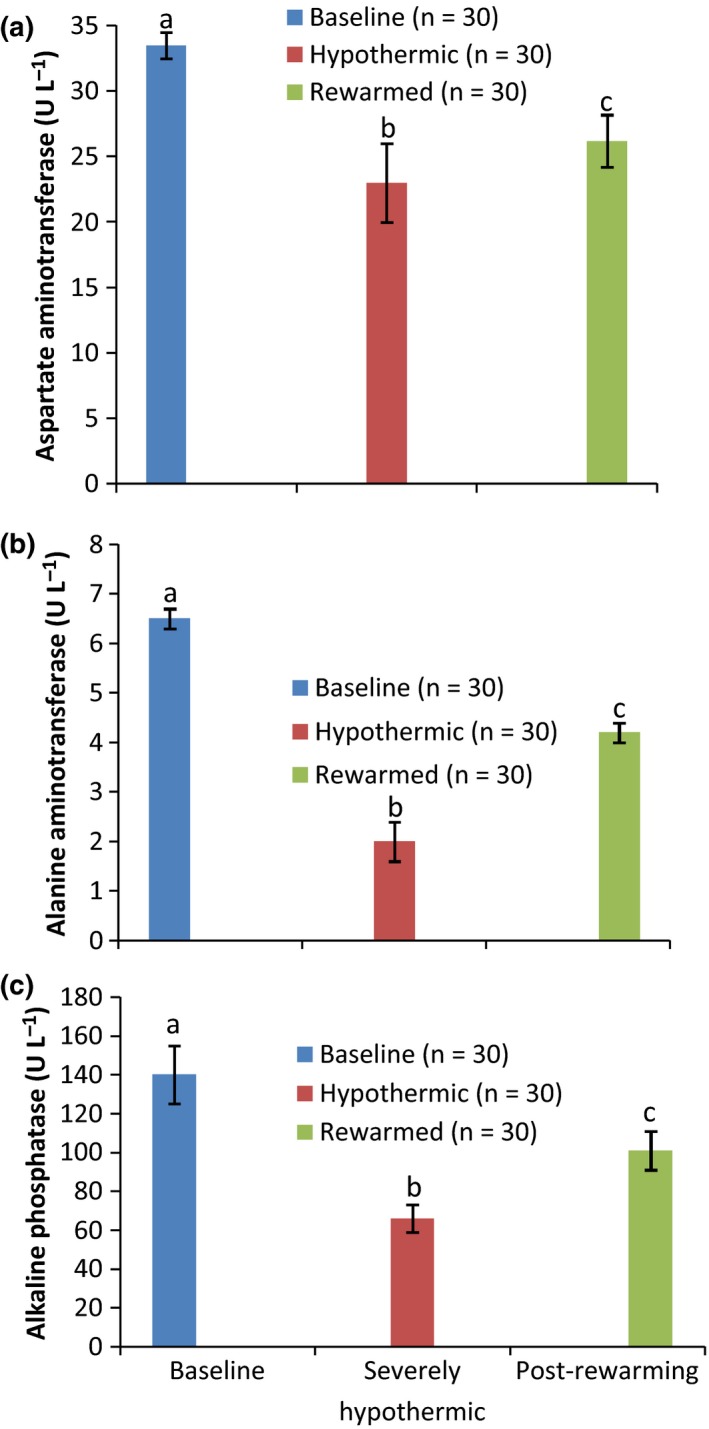
Activities of serum enzymes in normal, severely hypothermic and rewarmed pullets.

The biochemical results obtained from the severely hypothermic pullets on admission to the clinic and after 7 h of active external rewarming in comparison to baseline values are shown in Figs 1, 2, 3. The results showed a significant (*P *<* *0.05) decrease in the concentrations of serum electrolytes of Na, Cl, P, Ca, K (Fig. 1a–d and f), metabolites (Fig. 2a,b) and activities of enzymes of AST, ALT and ALP (Fig. 3a–c) in all the hypothermic birds compared to baseline values. However, 7 h of active external rewarming restored all the variables measured to baseline values. Although the activities of the enzymes post rewarming were significantly (*P *<* *0.05) lower than the baseline (Fig. 2a–c), they were within the normal minimum values. The bicarbonate (HC0_3_) concentrations were not affected by hypothermia or rewarming (Fig. 1e).

Weekly evaluation of the behavioural, CBT and biochemical values of the pullets for a period of 4 weeks after rewarming showed no deviation from the normal values, and there were no signs of any illness in all the birds.

## Discussion

The physical appearance and behaviour of the hypothermic birds presented to the clinic were similar to the behaviour in humans and other animals under different degrees of accidental hypothermia (Mallet 2002; Tomas 2011). Thus, on the basis of CBT and physical appearance of the birds in this study, accidental hypothermia was classified as severe hypothermia (CBT below 28°C).

Preliminary investigation has shown that poultry farmers and transporters discard severely hypothermic birds during transportation on the assumption that they are dead because they appear lifeless, motionless, with rigid bodies that are cold to the touch.

The cold feeling of the body to touch observed in the hypothermic pullets admitted to the clinic was due to the direct effect of protracted contact of the body with rain water and wind chill, exacerbated by the soaking wet feathers. These stimuli could have caused vasoconstriction, increased blood viscosity and decreased circulation of blood to the periphery so as to conserve heat. In addition, the cold rain water, apparently, drained heat from the body of the birds, which predisposed the body periphery to cold, perceived when touched.

The state of lifelessness and unconsciousness observed in the birds demonstrated the adverse effect of the cold on the cerebral hemisphere. At such low CBT, the cerebral neurological impairments are clinically first manifested in humans by coma and unconsciousness (Mallet 2002). These neurological changes are basically due to reduction in cerebral blood flow, cerebrovascular auto‐regulation and increase in cerebral ischaemia, when CBT falls below 30°C (Mallet 2002; Moore *et al*. 2011), as recorded in the severely hypothermic birds in this study.

The muscle and joint rigidity observed in severely hypothermic birds in this study were, apparently, due to impairment of peripheral nerve conduction induced by the cold stimuli, which may result in a gradual and progressive decline in conduction velocity, as reported in humans (Schmutzhard *et al*. 2012). The synaptic delay time may be prolonged as the neuromuscular junction cools, which may also inhibit muscle contraction, accompanied by hyporeflexia (Mallet 2002; Soleimanpour *et al*. 2014).

The CBT of 29 ± 0.4°C recorded in the birds admitted to the farm clinic suggested that the birds were severely hypothermic. The result is similar to the finding of Minka & Ayo (2016), who recorded a decrease of 8–10°C in CBT of hypothermic pullets exposed to wet‐cold weather transportation conditions.

Although there are inconsistent findings on the effects of hypothermia on serum electrolytes in humans and on different animal species (Mallet 2002; Kess & Polderman 2009), this study showed that accidental hypothermia induced hypocalcaemia, hypokalaemia, hypomagnesaemia, hypophosphotaemia, hyponatraemia and hypochloridaemia. This finding may be due to an increase in renal excretion of electrolytes, changes in membrane permeability and an influx of potassium into the cells caused by cold effect, which may predispose the pullets to electrolyte depletion (Mallet 2002; Scaravilli *et al*. 2012).

The hypokalaemia observed in this study was a good indicator of prognosis in the hypothermic pullets. This is because hyperkalaemia during hypothermia has a poor prognosis, and is considered an index of irreversible hypothermia (Scaravilli *et al*. 2012).

The decrease in the activities of AST, ALT, ALP and CRT observed in severely hypothermic pullets may be as a result of downward regulation in muscle and liver cells due to cold effect. Similar effect of cold on down‐regulation of activities of plasma enzymes was reported in animals and broilers exposed to different ambient temperatures in the temperate region (Chen *et al*. 2011). The downward regulation of the activities of the enzymes was primarily due to reduction in muscle cell membrane function and permeability as cold stress persisted (Mitchell & Sandercock 1995). The result of this study was in contrast with previous finding obtained in other hypothermic patients, shown to have mild or moderate elevated activities in serum enzymes of AST, ALT, CRT and ALP (Connolly & Worthley 2000). The reasons for these differences may be due to variation in species and the degree of hypothermia. However, the concentration of electrolytes and activities of the enzymes measured after rewarming demonstrated a gradual and successful return to baseline concentrations.

Rewarming is a delicate phase of therapeutic intervention in hypothermia, just as hypothermia itself is a very complex pathophysiological process. Human studies are limited to mild hypothermia because of ethical concerns. Based on the three rewarming techniques (passive external, active external and active internal) proposed for therapeutic hypothermia in humans and animals (European Resuscitation Council 2010; Brown *et al*. 2012; Minka & Ayo 2016), in this study, active external rewarming was employed. During the rewarming, emphasis was focused on the areas of the body with considerable vascular bed, particularly under the wings and chest. This approach, apparently, improved the gradual circulation of warm blood to the core organs, and prevented the restoration of cold peripheral venous blood; thus, preventing the outcome of ‘afterdrop’, the observation that as some patients were rewarmed, their core temperature continues to decline (Hunter *et al*. 1999). The slow pace of rewarming employed in this study, which lasted for 7 h, allowed for the gradual and safe recovery of CBT to normothermia. The result agreed with previous findings in humans (Moore *et al*. 2011) and animals (Bate *et al*. 1992; Wentworth *et al*. 2009; Tomas 2011; Minka & Ayo 2016), which demonstrated that several hours to many days of therapeutic rewarming are required for CBT to normalize.

The finding that the birds were able to stand, walk, preen, dust bath, eat and drink after about 7–10 h of active external gradual rewarming suggests that the normal and feeding behaviours of the birds were restored. The reason for the delay in the manifestation of the behaviours may be because there was little or no energy left in the pullets to perform these behaviours. The pullets may have prioritized their demand from these behaviours to those associated with heat conservation to maintain their normal CBT and other biochemical values.

The positive outcome of 7 h of rewarming the pullets suggests that chickens have a better prognosis in response to active external rewarming techniques compared to humans and other animal species, in which ‘afterdrop’ has been reported to be common and fatal in patients that were rewarmed (Connolly & Worthley 2000; Scaravilli *et al*. 2012). This study confirmed the tedious and time‐consuming task of rewarming technique, as reported by almost all researchers in this field (Kess & Polderman 2009; Moore *et al*. 2011). To the best of our knowledge, this study is one of the first reports to evaluate the behavioural and biochemical responses of severely hypothermic birds to rewarming treatment.

There is paucity of information in the available literature on the outcome of activities of blood enzymes and electrolyte concentrations in poultry, subjected to accidental hypothermia and rewarming that may warrant comparison. The present result may also be of value for human doctors in the area of severe hypothermia research where ethical concern has hampered studies. The chicken may serve as a good laboratory animal model for studying the pathophysiological mechanism of severe hypothermia and its rewarming outcome.

## Source of funding

This study was not funded by any granting or external agency.

## Conflict of interest

The authors declare that they have no financial or personal relationship(s), which may have inappropriately influenced them in writing this paper.

## Contributions

N.S.M was the project leader and clinician who rewarmed the birds. He supervised all procedures, provided logistics, collected and prepared all samples and data. J.O.A interpreted the results, provided an initial draft of the manuscript, statistically analysed the results and wrote the manuscript to standard. All authors reviewed and agreed the content of the final manuscript.
